# Attribution of Mental States in Glossolalia: A Direct Comparison With Schizophrenia

**DOI:** 10.3389/fpsyg.2020.00638

**Published:** 2020-04-15

**Authors:** Szabolcs Kéri, Imre Kállai, Katalin Csigó

**Affiliations:** ^1^Nyírõ Gyula Hospital, National Institute of Psychiatry and Addictions, Budapest, Hungary; ^2^Department of Cognitive Science, Budapest University of Technology and Economics, Budapest, Hungary; ^3^Department of Physiology, University of Szeged, Szeged, Hungary; ^4^Department of Psychiatry, University of Debrecen, Debrecen, Hungary

**Keywords:** glossolalia, schizophrenia, mentalization, Animated Triangle Test, Reading the Mind in the Eyes Test, spirituality

## Abstract

Glossolalia (“speaking in tongues”) is a rhythmic utterance of pseudo-words without consistent semantic meaning and syntactic regularities. Although glossolalia is a culturally embedded religious activity, its connection with psychopathology (e.g., psychotic thought disorder and altered mental state attribution/mentalization) is still a matter of debate. To elucidate this issue, we investigated 32 glossolalists, 32 matched control participants, and 32 patients with schizophrenia using the Animated Triangle Test (ATT) and the Reading the Mind in the Eyes Test (RMET). The ATT can detect hypo- and hypermentalization using animations of two moving triangles. Healthy adults describe these as random movements (e.g., bouncing), willed actions (e.g., playing), or they mentalize (e.g., tricking). We found that glossolalists provided more mentalizing descriptions in the ATT random and intentional movement animations relative to the control participants. They also recognized more mental states in the RMET than the controls. None of them had a diagnosis of mental disorders. In contrast, patients with schizophrenia hypermentalized only in the ATT random movement condition, whereas they showed hypomentalization in the ATT intentional movement condition and in the RMET relative the control subjects. Hypermentalization in the ATT positively correlated with intrinsic religiosity in the glossolalia group. In conclusion, our results demonstrated a substantial difference in the mentalizing ability of glossolalists (generalized hypermentalization) and patients with schizophrenia (both hypo- and hypermentalization).

## Introduction

Glossolalia is a poetic-rhythmic utterance of pseudo-words without constant semantics and syntax. It is regularly produced in a religious and spiritual context with particular reference to charismatic Christian and Pentecostal communities (Goodman, 1972; Mills, 1986; Holm, 1991; Cartledge, 2002). The term glossolalia stems from a Greek phrase used in the Acts and 1 Corinthians in the New Testament [γλωσσoλαλíα, *glossa* (tongue or language) and *laleo* (speak or talk), “speaking in tongues”]. The default cultural interpretation of this phenomenon delineates a “heavenly language of the spirit” accessible only to the gifted ones. Glossolalists often report an intentional or spontaneous suspension of will to convey divine messages and prophecies. They tend to perceive an external locus of control, a conviction that extrapersonal forces possess control over their life (Coulson and Johnson, 1977). These experiences are a part of intrinsic religiosity, which characterizes religious motivation and commitment (e.g., the personal experience of a divine, supernatural, or higher power; religious beliefs as a fundamental and a holistic approach to life; carrying religion over into all other dealings) (Koenig et al., 1997; Hood et al., 2009; Koenig and Büssing, 2010).

In a seminal cross-cultural study, Samarin (1972) demonstrated that glossolalic speech is not a random and disorganized production of sounds: it is characterized by specific accent, intonation, and word-like and sentence-like units consisting of syllables, consonants, and vowels, which are not radically different from the original language of the speaker (De Peza, 1996). Glossolalia is a collectively accepted form of religious activity in contrast to language anomalies outside a cultural context (Chouiter and Annoni, 2018).

Three broad hypotheses are attempting to explain the origin and causes of glossolalia, emphasizing its relationship with psychopathology (i.e., disorganized thinking and speech in psychotic disorders) (Cutten, 1927; Goodman, 1973; Samarin, 1973; Spencer, 1975; Brende and Rinsley, 1979; Hempel et al., 2002; Francis and Robbins, 2003; Reeves et al., 2014), altered states of consciousness (Goodman, 1972; Kavan, 2004), and social learning (Kildahl, 1972; Malony and Lovekin, 1985; Spanos et al., 1986; Koic et al., 2005; Johnson, 2010). Despite initial research linking glossolalia to schizophrenia, mood disorders, and dissociative disorders (Cutten, 1927; Goodman, 1973; Samarin, 1973; Spencer, 1975; Brende and Rinsley, 1979; Hempel et al., 2002; Francis and Robbins, 2003; Reeves et al., 2014), there is scarce and inconsistent evidence that socially embedded glossolalia is an abnormal phenomenon (Castelein, 1984; Grady and Loewenthal, 1997; Johnson, 2010). For example, it has been shown that schizophasia (grossly disorganized and incoherent speech in schizophrenia and other psychotic disorders) is linguistically distinguishable from glossolalia (Samarin, 1973). Also, glossolalists seem to display lower rates of depression (Spanos and Hewitt, 1979), less neuroticism, and higher emotional stability as compared to non-glossolalists with a similar cultural and religious background (Francis and Robbins, 2003), which is against the psychopathology hypothesis.

However, social cognitive functions have not been compared in glossolalia and psychotic disorders. The assessment of theory of mind (ToM) and mentalization (the attribution of mental states, including intentions, beliefs, desires, and complex social emotions) is of particular relevance (Carruthers and Smith, 1996; Bateman and Fonagy, 2010). Experiencing the presence of a higher power and conveying divine messages are imaginative, symbolic, and culturally meaningful acts, requiring the attribution of mental states to invisible agents (gods, ghosts, angels, and other culturally embedded spiritual actors) (Boyer, 2001; Newberg et al., 2002, 2006; Schjoedt et al., 2009). Heightened mentalizing activity may be a cornerstone of religious and spiritual cognition, which may be enhanced in individuals practicing glossolalia who experience direct access into the mental states of imaginary beings.

Concerning the psychopathological model of glossolalia, it is notable that altered mentalization is a well-replicated feature of schizophrenia (Abu-Akel and Bailey, 2000; Frith, 2004; Brune, 2005; Bora and Pantelis, 2013; Bliksted et al., 2016, 2019; Martinez et al., 2019). Some patients exhibit a weakened attribution of mental states (hypomentalization), resulting in social isolation and inflexible communication, which is reminiscent of that seen in individuals with autism-spectrum disorders (Martinez et al., 2019). Hypomentalization is also related to formal thought disorder and impaired comprehension with a particular reference to clause embedding (Cokal et al., 2019). However, patients with schizophrenia also tend to interpret neutral and random social interactions as if they were driven by intentional agents (hypermentalizing), which may lead to altered causal attribution and abnormal belief formation (Abu-Akel and Bailey, 2000; Frith, 2004). Under the circumstances characterized by stress, social threat, and anomalous perceptual experiences, hypermentalization may form the basis for paranoid ideas (Freeman and Garety, 2014; Ballespi et al., 2019). Intriguingly, hypo- and hypermentalization can simultaneously occur in the same patient who experiences social difficulties, impaired language comprehension, and paranoid thoughts at the same time (Bliksted et al., 2016, 2019). Thus, inadequate hypermentalization is a possible common feature of schizophrenia and glossolalia, resulting in different cognitive and behavioral outcomes.

**FIGURE 1:**
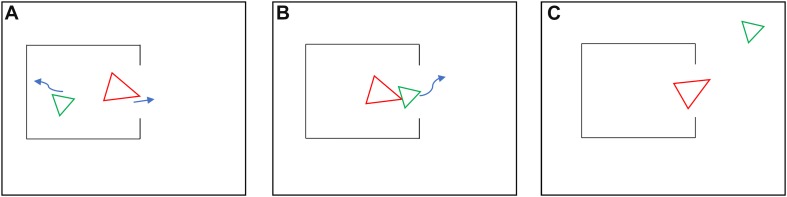
Illustration of the Animated Triangle Test (ATT) showing characteristic Frith-Happé animations and typical mentalizing descriptions. **(A)** Mother tries to motivate her child to go outside. **(B)** Child does not want to go out. **(C)** Child exploring and playing outside, and mother watches her.

The purpose of the present study was to compare mentalization in individuals with glossolalia and patients with schizophrenia by using the Animated Triangle Test (ATT) (Abell et al., 2000; Castelli et al., 2000, 2002; Martinez et al., 2019). This test is suitable for the detection of both hypo- and hypermentalization by implementing animations of two triangles that move around the screen (Figure 1). Healthy adults describe the actions of the triangles as random movements (e.g., bouncing or wandering), goal-directed willed actions (e.g., fighting or playing), or observers mentalize and attribute mental states to the triangles (e.g., tricking or wanting to hide). In hypomentalization, there are fewer mentalizing descriptions, whereas, in hypermentalization, observers attribute mental states even to random movements (Eddy and Cavanna, 2015; Bliksted et al., 2019). We also used the Reading the Mind in the Eyes Test (RMET), which assesses mentalization driven by the recognition of complex social emotions from facial expression (eye-regions depicted in photographs) (Baron-Cohen et al., 2001). The Social Cognition Psychometric Evaluation (SCOPE) project recommended the application of the RMET because of its practical features in clinical settings, acceptable internal consistency, good criterion validity, and its correlation with rehabilitation outcome (Pinkham et al., 2016, 2018).

We hypothesized that individuals with glossolalia show hypermentalization in the absence of mental illness, depressive-anxious features, or enhanced schizotypal traits. In contrast, patients with schizophrenia are characterized by both hypo- and hypermentalization, together with a decreased accuracy in the description of the ATT animations (Bliksted et al., 2016, 2019). We also hypothesized that hypermentalization is related to the subjective experience of intrinsic religiosity. We also studied the association between mentalization and organized/private religious activity, as measured by the Duke University Religiosity Index (DUREL) (Koenig et al., 1997; Koenig and Büssing, 2010).

## Materials and Methods

### Participants

We recruited 32 glossolalists and 32 control volunteers who did not practice glossolalia from local Pentecostal and charismatic communities. The criteria of glossolalia were established according to Chouiter and Annoni (2018): (1) few or no recognizable words with semantic meaning except for Biblical phrases; (2) similar phonemic properties to the language of the speaker; (3) small phoneme numbers, accelerated speech, and altered accent; (4) phonetic pulses begin with a consonant, bars are of equal length, primary accent on the first pulse of each bar, and sentences with similar lengths; (5) an ability to speak “tongues” or ordinary language; and (6) absence of neurological illness.

The third group of participants consisted of 32 outpatients with schizophrenia who were demographically similar to the glossolalia and non-glossolalia group, including education, IQ, social-economic status, and religious activity (Table 1). The patients were recruited from a large and heterogeneous outpatient sample at the National Institute of Psychiatry and Addictions (Budapest, Hungary). The patient group included highly functioning and clinically stable individuals in order to minimize the confounding effect of the chronic disease process. None of the patients received electroconvulsive therapy. All participants were Hungarian Caucasians. Written informed consent was obtained from each volunteer. The study was approved by the National Medical Research Council (ETT-TUKEB 18814, Budapest, Hungary). All research was performed following relevant guidelines and regulations.

**TABLE 1:** Characteristics of the participants.

	**Glossolalists**	**Non-glossolalists**	**Patients with**
	**(*n* = 32)**	**(*n* = 32)**	**schizophrenia (*n* = 32)**
Gender (male/female)	20/12	20/12	20/12
Age (years)	31.4 (5.1)	32.3 (6.2)	31.9 (4.7)
Education (years)	11.3 (3.6)	11.4 (3.9)	11.0 (4.8)
Wechsler Adult Intelligence Scale – IV	101.2 (10.6)	102.0 (11.0)	99.5 (12.2)
Hollingshead Four Factor Index (socioeconomic status)	32.6 (7.5)	32.4 (8.0)	31.9 (6.3)
Hamilton Depression Rating Scale (normal: 0–7 points)	6.3 (2.4)	6.5 (3.3)	8.2 (7.1)
**Oxford-Liverpool Inventory of Feeling And Experiences (O-LIFE)**			
Unusual experiences (0–12 points)	2.8 (1.6)	2.9 (1.9)	6.8(2.4)*
Introvertive anhedonia (0–10 points)	2.4 (0.9)	2.2 (1.1)	4.5(2.6)*
Cognitive disorganization (0–11 points)	4.1 (2.0)	4.4 (1.0)	6.0(3.0)*
Impulsive non-conformity (0–10 points)	2.5 (0.7)	2.1 (0.8)	3.7(1.0)*
**Duke University Religiosity Index (DUREL)**			
Organized religious activity (1–5 points)	3.9 (1.5)	3.8 (1.4)	3.4 (2.6)
Non-organized (private) religious activity (1–5 points)	3.1 (1.1)	3.6 (1.5)	3.2 (1.9)
Intrinsic religiosity (1–5 points)	3.4 (1.0)	3.9 (1.4)	3.5 (1.2)
**Positive and Negative Syndrome Scale**			
Positive symptoms	–	–	12.5 (6.8)
Negative symptoms	–	–	14.1 (7.2)
Generalized symptoms	–	–	41.1 (13.6)
**Antipsychotic medications**			
Antipsychotic chlorpromazine equivalent dose (mg/day)	–	–	378.6 (297.0)
Antipsychotic type (second-/first-generation)	–	–	26/5
**Other illness parameters**			
Duration of illness (years)			4.0 (2.6)
Number of hospitalizations			2.1 (1.5)

Data are mean (standard deviation) except for gender and antipsychotic type. All patients with schizophrenia received stable antipsychotic medications at the time of testing (olanzapine, quetiapine, risperidone, aripiprazole, zuclopenthixol, and flupenthixol). *Higher O-LIFE values in the schizophrenia group relative to the other two groups (*p*s < 0.05).

### General Assessment

All participants received the structured clinical interview for DSM-5 (Diagnosis and Statistical Manual of Mental Disorders – 5) disorders to confirm the diagnosis of schizophrenia and to exclude mental disorders in the glossolalia and non-glossolalia group (First et al., 2016). None of the participants in the glossolalia and non-glossolalia group meet the criteria of mental disorders. In addition to the DSM-5 interview, the following tools were used to characterize the participants: Wechsler Adult Intelligence Scale-IV (WAIS-IV) (Wechsler, 2008), Hollingshead Four-Factor Index of Socioeconomic Status (SES) (Hollingshead, 1975), Hamilton Depression Rating Scale (HAM-D) (Hamilton, 1960), and the Positive and Negative Syndrome Scale (PANSS) (Kay et al., 1987) (patients only) (Table 1).

### Oxford-Liverpool Inventory of Feelings and Experiences

The Oxford-Liverpool Inventory of Feelings and Experiences (O-LIFE) assesses schizotypal personality traits (Mason and Claridge, 2006). The self-report questionnaire consists of yes/no items organized in four subscales: Unusual Experiences (30 items, positive schizotypy: hallucination-like experiences, perceptual aberrations, and magical thinking; internal consistency: α = 0.85), Cognitive Disorganization (24 items, disorganized schizotypy: loosened associations, poor attention, and decision-making, social anxiety; internal consistency: α = 0.83), Introvertive Anhedonia (27 items, negative schizotypy: physical and social anhedonia, avoidance of intimacy, social withdrawal; internal consistency: α = 0.77), and Impulsive Non-conformity (23 items, impulsive schizotypy: impulsive, antisocial, eccentric, and aggressive tendencies; internal consistency: α = 0.70) (Table 1).

### Duke University Religiosity Index

The modified DUREL is a self-report measure consisting of five items (Koenig and Büssing, 2010). The instrument assesses organized religious activity [one item: attendance of church and religious gatherings; never (1) – more than once a week (6)], non-organized religious activity [one item: prayer, meditation, or Bible study; rarely or never (1) – more than once a day 6)], and intrinsic (subjective) religiosity [three items: experiencing the Divine in life, religious beliefs lie behind the whole approach to life, and carrying religion over into all other dealings in life; definitely not true (1) – definitely true (5)]. The DUREL exhibited excellent internal consistency (α = 0.89) (Table 1).

### Animated Triangle Test

Participants viewed brief video clips on the computer screen depicting two triangles (Frith-Happé animations) (Figure 1; Abell et al., 2000; Castelli et al., 2000, 2002). There were two conditions. In the random (non-intentional) condition, the triangles moved arbitrarily (e.g., bouncing or flotation). In the intentional condition, the movement of the triangles mimicked social interactions that made the viewers feel that the triangles had mental states and influenced each other (e.g., tricking or playfulness). There were eight animations (four random and four intentional, duration: 38–41-s each). When an animation clip terminated, participants reported what they thought was happening during the short movies. Two behavioral scientists or psychologists, who were not aware of the aim of the study and the status of the participants (glossolalists, non-glossolalists, and schizophrenia), assessed the answers and calculated the mean scores. There were two aspects of scoring: intentionality (degree of mental state attribution, range 0–5) and accuracy (how exact was the description, range 0–3; Castelli et al., 2000, 2002). The inter-rater agreement between the two assessors was high (intentionality for random animations: κ = 0.79, *Z* = 4.9, *p* < 0.001; intentionality for non-random animations: κ = 0.81, *Z* = 9.6, *p* < 0.001; accuracy for random animations: κ = 0.78, *Z* = 4.8, *p* < 0.001; accuracy for non-random animations: κ = 0.82, *Z* = 9.5, *p* < 0.001).

### Reading the Mind in the Eyes

Participants saw 36 photographs depicting eye-regions of faces of actors and actresses. Each photograph appeared on separate slides. Eye-regions expressed complex social emotions and mental states (e.g., playful, terrified, and joking). Without any time-pressure, participants choose which of four words (one target and three foils) best described the mental state of the actor or actress on the photographs. The dependent measure was the number of correct responses (Baron-Cohen et al., 2001).

### Statistical Analysis

We used STATISTICA 13.1 (Tibco, Palo Alto) software package for data analysis. First, we conducted Kolmogorov–Smirnov and Levene’s tests to evaluate data distribution and the homogeneity of variance, respectively. Given that the data were normally distributed, and the variances were homogeneous (*p*s > 0.2), we used parametric statistical tests. Behavioral measures were entered into analyses of variance (ANOVAs) to investigate the main effect of group (glossolalia, non-glossolalia, and schizophrenia), test conditions (random and intentional movement), and the interaction between the group and test conditions. The ANOVAs were followed by Tukey’s Honestly Significant Differences (HSD) *post hoc* tests. The IQ and SES were covariates in the ANOVAs. Pearson’s product moment correlation coefficients were calculated between the ATT/RMET data and the demographic and clinical parameters. Correlation analyses were also corrected for IQ and SES (partial correlations). Multiple regression analyses were conducted to determine the predictors of religiosity. Demographic parameters were compared with one-way ANOVAs, Student’s *t*-tests (two-tailed), and chi-square tests. The level of statistical significance was set at alpha <0.05 (corrected for multiple comparisons with the Benjamini–Hochberg method).

## Results

### Performance on the ATT

**FIGURE 2:**
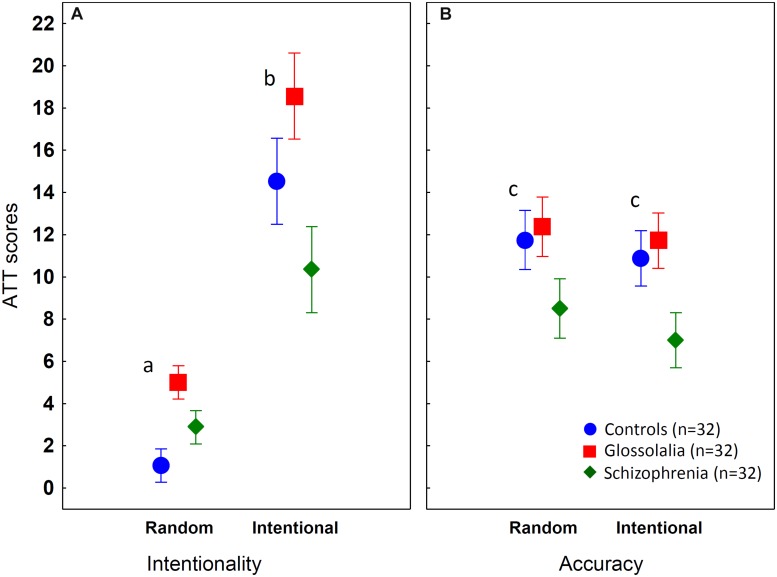
Results from the Animated Triangle Test (ATT) (**A** - intentionality and **B** - accuracy). The graphs show mean values, the error bars indicate 95% confidence intervals. ^a^Controls < Schizophrenia < Glossolalia; ^b^Schizophrenia < Controls < Glossolalia (*p*s < 0.05); ^c^Schizophrenia < Controls = Glossolalia (all significant differences at *p*s < 0.05, Tukey’s HSD tests).

First, we compared the ATT performance (attribution of intentionality to the stimuli) of glossolalists, patients with schizophrenia, and matched control subjects (Table 1). The ANOVA conducted on the ATT scores revealed a significant main effect of group [*F*(2,93) = 20.44, *p* < 0.001, η*^2^* = 0.31], test condition [random vs. intentional) (*F*(1,93) = 401.63, *p* < 0.001, η*^2^* = 0.81], and a two-way interaction between group and test condition [*F*(2,93) = 12.34, *p* < 0.001, η*^2^* = 0.21]. Individuals practicing glossolalia detected more intentionality in the random and intentional animations than the non-glossolalia control subjects (*p*s < 0.05) (Figure 2A). Patients with schizophrenia also scored above the control participants in the random animations (*p* < 0.05), but in the intentional movement animations, they achieved a lower score relative to the controls (*p* < 0.01). Finally, a head-to-head comparison between individuals with glossolalia and patients with schizophrenia indicated no significant difference in the random condition (*p* > 0.1), whereas, in the intentional movement animations, the patients performed worse than the glossolalia group (*p* < 0.001) (Figure 2A). The above-described results remained the same when IQ and SES were included in the analysis as covariates.

### Accuracy on the ATT

When we analyzed the accuracy of the descriptions related to the ATT animations, we found significant main effects of group [*F*(2,93) = 12.02, *p* < 0.001, η*^2^* = 0.21] and test condition (random vs. intentional) [*F*(1,93) = 27.9, *p* < 0.001, η*^2^* = 0.23). The two-way interaction between group and condition did not reach the level of statistical significance (*p* > 0.1). There were no significant differences between the glossolalia and the non-glossolalia group (*p* > 0.5). In contrast, patients with schizophrenia were less accurate than glossolalists and control participants in all conditions (*p* < 0.05) (Figure 2B). The results of the accuracy analysis were the same when IQ and SES were included as covariates.

### Performance on the RMET

Regarding mentalization related to the recognition of complex social emotions, the ANOVA performed on the RMET scores indicated a significant main effect of group [*F*(2,93) = 20.80, *p* < 0.001, η*^2^* = 0.31]. Tukey’s HSD tests revealed that glossolalists outperformed controls [*M* (control) = 23.6, SD = 4.9; *M* (glossolalia) = 26.2, SD = 4.2; *p* < 0.05] and patients with schizophrenia [*M* (schizophrenia) = 19.1, SD = 4.3; *p* < 0.001] who were impaired relative to the control group (*p* < 0.001).

### Relationship Between Mentalization and Religiosity

**FIGURE 3:**
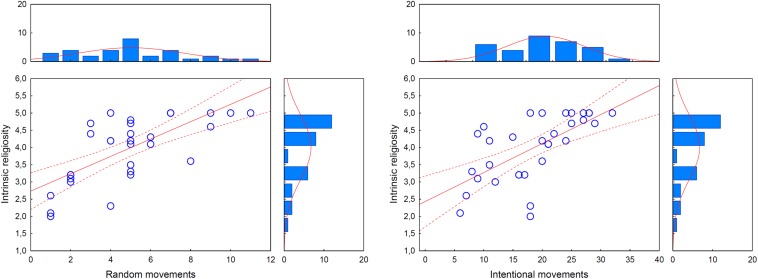
Correlations between intrinsic religiosity and intentionality attributed to random movements (*r* = 0.71, *p* < 0.01) and intentional movements (*r* = 0.65, *p* < 0.01) on the Animated Triangle Test (ATT). Dotted lines indicate 95% confidence intervals. The graphs on the top and on the right indicate the distribution of ATT scores and intrinsic religiosity scores, respectively.

In the glossolalia group, higher scores on the ATT random and intentional conditions were associated with higher DUREL intrinsic religiosity scores [*r*(random) = 0.71, *r*(intentional) = 0.65, *p*s < 0.01) (Figure 3]. Both random and intentional ATT values predicted intrinsic religiosity (random: *b*^∗^ = 0.51, *t*(29) = 3.76, *p* < 0.01; intentional: *b*^∗^ = 0.39, *t*(29) = 2.88, *p* < 0.05), altogether explaining the 58.1% of the variance in the DUREL intrinsic religiosity score [*F*(2,29) = 22.52, *p* < 0.001]. In the other two groups, we found no significant relationships between ATT and DUREL scores. There were no significant correlations between ATT and DUREL organized/personal religiosity scores in either group. The RMET did not correlate with the DUREL scores (organizational, private, and intrinsic components) in either group (−0.3 < *r*s < 0.3, *p*s > 0.1).

## Discussion

Our results confirmed the hypothesis that glossolalists display hypermentalization. Specifically, they more frequently attributed intentions to randomly moving objects on the ATT as compared to non-glossolalists with similar demographic and religious features. In this respect, glossolalists and patients with schizophrenia were similar. However, individuals practicing glossolalia were also highly sensitive to intentionality when the animated objects interacted with each other to mimic social behavior in the ATT. Glossolalists also recognized more complex mental states in the RMET than the controls. General hypermentalization is a critical feature that distinguished glossolalists from patients with schizophrenia who achieved lower scores on the ATT intentional condition and the on RMET relative to the control volunteers. In other words, patients with schizophrenia showed hypomentalization in the ATT intentional condition and in the recognition of facial expression of complex mental states, whereas glossolalists hypermentalized in all test conditions.

The hypermentalizing feature of schizophrenia was inadequate and possibly maladaptive because the patients exclusively interpreted random movements as if these were intentional: they detected social salience in a non-salient situation (Kapur, 2003). In glossolalia, however, we also observed enhanced mentalizing sensitivity in the intentional condition, which may be an adaptive feature. For the interpretation of the results, it is essential to emphasize that the differences summarized above were demonstrated in groups similar in demographic features and religiosity, which may have an impact on mentalization. IQ and SES did not explain group differences because the groups did not differ in these measures, and the results remained the same when these parameters were included as covariates in the statistical analyses.

Beyond the characterization of mentalization, an important finding of our study is that individuals with glossolalia did not meet the criteria of DSM-5 disorders, and their depression, anxiety, and schizotypy scores did not differ from the values of the non-glossolalist volunteers. Therefore, our results are against the hypothesis that glossolalia is a consequence of psychopathology (Cutten, 1927; Goodman, 1973; Samarin, 1973; Spencer, 1975; Brende and Rinsley, 1979; Hempel et al., 2002; Francis and Robbins, 2003; Reeves et al., 2014). However, we did not conduct a detailed personality assessment, and we cannot make a conclusion on individual differences between glossolalists and non-glossolalists. The relationship between psychopathology and glossolalia should be explored in a larger representative sample.

From a broader perspective, our findings provide further evidence that enhanced mentalization is an essential component of religious and spiritual cognition. The leading cognitive theory of religion focuses on the concept of hyperactive agent detection device (HADD) as an evolutionary ancient mental faculty serving social cognition (Barrett, 2000; Boyer, 2001; Atran, 2002). According to the HADD model, humans are hardwired to impute intentions in salient situations. These situations do not necessarily include human beings and animate things: mental states and intentions can be attributed to inanimate objects and physical forces. People attribute magical causes to existential, natural, economic, and political events to create meaning and understanding (e.g., richness, health, and blooming are presents, whereas war, illness, and disaster are punishment from gods, spirits, devil, and other higher powers) (Barrett, 2000; Boyer, 2001; Atran, 2002). In adolescents with autism-spectrum conditions, neurotypical student samples, and Canadian and American national samples, Norenzayan et al. (2012) demonstrated a link between autistic traits and reduced belief in God, and mentalizing capacities mediated this relationship. It is notable that the authors controlled the mediation analysis for well-known personality features related to religiosity (systemizing, conscientiousness, and agreeableness) (Norenzayan et al., 2012). However, subsequent studies failed to demonstrate a significant relationship between mentalization and religious belief, and emphasized other potential mediators (e.g., core ontological confusions, moral concern, analytical thinking, and credibility enhancing displays) (Lindeman et al., 2015). These results challenge the theoretical view that enhanced perception of agency can explain the emergence of religious and spiritual beliefs. In the present study, we could refine this hypothesis by demonstrating a specific relationship between glossolalia and hypermentalizing. Moreover, increased mentalization, primarily when intentionality was attributed to random movements, was associated with intrinsic religiosity, which includes the feeling of immersion in the thoughts, plans, and intentions of supernatural beings. Therefore, agency detection may be related to a circumscribed aspect of religious cognition (i.e., the experience of contact and communication with supernatural beings) and not to religious beliefs in general.

The present study is not without limitations, and our initial results on glossolalia and social cognition raise several questions for future studies. First, the sample size was small, and the study was underpowered. This limitation is explained by the uniqueness and rarity of the sample. Individuals with glossolalia are often reluctant to participate in research studies. Therefore, the study must be replicated and extended in a large and representative sample. Second, it is unclear how specific linguistic features are related to mentalization. This issue necessitates a multifaceted assessment of mental state attribution with both visual and verbal tests, together with the linguistic analysis of glossolalic speech. Third, we used only two tests to measure mentalization, which is a complex psychological construct comprising distinct components. Moreover, the impact of enhanced mentalization on daily functioning and quality of life should be elucidated. The ecological validity of these experimental procedures is not clearly understood (Byom and Mutlu, 2013). Forth, the present study failed to address the putative link between mentalization and personality traits, which is crucial to better understand the interplay among religiosity, spirituality, individual differences, and social-cognitive mechanisms.

Furthermore, the issue of culture, gender, race, ethnicity, social-economic status, and other demographic measures are of prominent importance in glossolalia research. In this respect, the critical feature of the present study was that we assessed a culturally, racially, and ethnically homogeneous population. All participants were Caucasian with Hungarian origin. Therefore, our data do not add new information regarding the diversity of ethnocultural context of glossolalia.

When interpreting the present results, a potential selection bias must be taken into consideration. Specifically, it seems to be evident that patients with schizophrenia, who experience a potentially deteriorating course of illness, display a poor performance on mentalization tasks as compared to glossolalists and controls without a mental disorder. However, we selected highly functioning outpatients who were not statistically different in education, IQ, social-economic status, and religiosity concerning the other two study groups. We observed mild negative symptoms in our schizophrenia sample (14.1 points on a 7–49 points negative symptom scale of PANSS; average rating for the seven symptoms: minimal, questionable, and subtle pathology, or the extreme end of the normal range; Kay et al., 1987). Also, patients with schizophrenia did not show a generalized impairment on the mentalization tests: in the ATT random movement condition, they outperformed the healthy control group. The second source of bias is that glossolalists were not recruited from the general community. Therefore, the results should be replicated and extended in a larger and less specific sample.

## Conclusion

In conclusion, culturally embedded glossolalia in religious settings can be present in the absence of profound psychopathology. Glossolalists exhibit generally enhanced mentalization, which is different from patients with schizophrenia who display a double pattern of hypo- and hypermentalization. Therefore, it has not been justified that glossolalia can be viewed as a form or a symptom of mental disorders.

## Data Availability Statement

The datasets generated for this study are available on request to the corresponding author.

## Ethics Statement

The studies involving human participants were reviewed and approved by the National Medical Research Council (ETT-TUKEB 18814, Budapest, Hungary). The patients/participants provided their written informed consent to participate in this study.

## Author Contributions

SK coordinated data collection and measurements, and wrote the first draft of the manuscript. SK and KC analyzed the data. IK performed the revision of the manuscript. All authors designed the study, reviewed and edited the final version of the manuscript, and approved the manuscript for publication.

## Conflict of Interest

The authors declare that the research was conducted in the absence of any commercial or financial relationships that could be construed as a potential conflict of interest.
